# Xpert-Ultra Assay in Stool and Urine Samples to Improve Tuberculosis Diagnosis in Children: The Médecins Sans Frontières Experience in Guinea-Bissau and South Sudan

**DOI:** 10.1093/ofid/ofae221

**Published:** 2024-05-02

**Authors:** Laura Moretó-Planas, Raman Mahajan, Lazro Fidelle Nyikayo, Yoanis Bedpinj Peter Ajack, Buai Tut Chol, Eltigani Osman, Mitchell Sangma, Apal Tobi, Jonathan Gallo, Evelize Biague, Ramiro Gonçalves, Mercè Rocaspana, Cándida Medina, Miguel Camará, Laurence Flevaud, Lisa C Ruby, Sabine Bélard, María José Sagrado, Israel Molina, Augusto E Llosa

**Affiliations:** Medecins Sans Frontières, Medical Department, Barcelona, Spain; Autonomous University of Barcelona, Faculty of Medicine, Barcelona, Spain; Medecins Sans Frontières, New Delhi, India; Medecins Sans Frontières, Malakal, Republic of South Sudan; Medecins Sans Frontières, Malakal, Republic of South Sudan; Medecins Sans Frontières, Juba, Republic of South Sudan; Medecins Sans Frontières, Juba, Republic of South Sudan; Medecins Sans Frontières, Nairobi, Kenya; National Tuberculosis Program, Ministry of Health, Juba, Republic of South Sudan; Medecins Sans Frontières, Bissau, Guinea-Bissau; Medecins Sans Frontières, Bissau, Guinea-Bissau; Medecins Sans Frontières, Bissau, Guinea-Bissau; Medecins Sans Frontières, Medical Department, Barcelona, Spain; Simão Mendes Hospital, Bissau, Guinea-Bissau; National Tuberculosis Program, Ministry of Health, Bissau, Guinea-Bissau; Medecins Sans Frontières, Medical Department, Barcelona, Spain; Institute of Tropical Medicine, University of Tübingen, Tübingen, Germany; German Centre for Infection Research (DZIF), Tübingen, Germany; Institute of Tropical Medicine, University of Tübingen, Tübingen, Germany; German Centre for Infection Research (DZIF), Tübingen, Germany; Medecins Sans Frontières, Medical Department, Barcelona, Spain; Infectious Disease Department, Vall d’Hebron Hospital, Barcelona, Spain; Medecins Sans Frontières, Medical Department, Barcelona, Spain

**Keywords:** LMIC, pediatric tuberculosis, stool, urine, Xpert-Ultra

## Abstract

**Background:**

More than half of childhood tuberculosis cases remain undiagnosed yearly. The World Health Organization recommends the Xpert-Ultra assay as a first pediatric diagnosis test, but microbiological confirmation remains low. We aimed to determine the diagnostic performance of Xpert-Ultra with stool and urine samples in presumptive pediatric tuberculosis cases in 2 high-tuberculosis-burden settings.

**Methods:**

This Médecins Sans Frontières cross-sectional multicentric study took place at Simão Mendes Hospital, Guinea-Bissau (July 2019 to April 2020) and in Malakal Hospital, South Sudan (April 2021 to June 2023). Children aged 6 months to 15 years with presumptive tuberculosis underwent clinical and laboratory assessment, with 1 respiratory and/or extrapulmonary sample (reference standard [RS]), 1 stool, and 1 urine specimen analyzed with Xpert-Ultra.

**Results:**

A total of 563 children were enrolled in the study, 133 from Bissau and 400 from Malakal; 30 were excluded. Confirmation of tuberculosis was achieved in 75 (14.1%), while 248 (46.5%) had unconfirmed tuberculosis. Of 553 with an RS specimen, the overall diagnostic yield was 12.4% (66 of 533). A total of 493 stool and 524 urine samples were used to evaluate the performance of Xpert-Ultra with these samples. Compared with the RS, the sensitivity and specificity of Xpert-Ultra were 62.5% (95% confidence interval, 49.4%–74%) and 98.3% (96.7%–99.2%), respectively, with stool samples, and 13.9% (7.5%–24.3%) and 99.4% (98.1%–99.8%) with urine samples. Nine patients were positive with stool and/or urine samples but negative with the RS.

**Conclusions:**

Xpert-Ultra in stool samples showed moderate to high sensitivity and high specificity compared with the RS and an added diagnostic yield when RS results were negative. Xpert-Ultra in stool samples was useful in extrapulmonary cases. Xpert-Ultra in urine samples showed low test performance.

**Clinical Trials Registration:**

NCT06239337

## Teaser

In a large cohort of presumptive pediatric tuberculosis cases from 2 high-burden tuberculosis settings, the Xpert-Ultra assay in stool samples had high diagnostic accuracy and an added diagnostic yield, while its performance in urine samples was low.

Despite being a preventable and treatable disease, tuberculosis continues to be an important cause of disease and death in children [1], especially among those living with human immunodeficiency virus (HIV) and/or malnutrition. More than half of childhood tuberculosis cases remain undiagnosed every year due to several factors, including a low rate of microbiological confirmation, the need for partly invasive procedures in young children, and low access to microbiological testing, including tuberculosis culture or the Xpert-Ultra assay, and to chest radiography [2–5]. Thus, in many contexts the diagnosis of tuberculosis in children is based solely on clinical presentation despite the poor specificity of clinical features, which ultimately contributes to the diagnostic gap.

Tuberculosis is the main comorbid condition and cause of death in people living with HIV, including children [6], and it is also a common comorbid condition along with severe malnutrition in tuberculosis-endemic environments. Malnutrition is an independent risk factor for both development of and death due to tuberculosis and is associated with more severe forms of the disease [7–9].

Although the reference standard for diagnosis is tuberculosis culture, it is often unavailable and requires a high turnaround time to be used routinely. Since 2013, the World Health Organization (WHO) recommends Xpert MTB/RIF (Cepheid) as first test for the diagnosis of tuberculosis in children [10, 11], considering the low sensitivity of smear microscopy [12]. An international expert panel established that for study purposes the case definition of “confirmed tuberculosis” included a positive result of a WHO-endorsed nucleic acid amplification test (such as Xpert MTB/RIF) [13–15].

Xpert MTB/RIF has been widely studied for pulmonary [16] and extrapulmonary [17–19] samples, as well as stool samples [20–24], which, being easily obtainable, are considered suitable complementary samples, especially for children unable to provide respiratory samples. Urine samples are not yet recommended, and most of the related studies focus on adults with HIV [25–27].

Cepheid developed the Xpert-Ultra assay to improve sensitivity of tuberculosis detection with a special focus on people living with HIV, children, and extrapulmonary forms [27–30]. The increase in sensitivity has been at the expense of lower specificity, especially among patients with a history of tuberculosis treatment. Stool proved to be a suitable sample for Xpert-Ultra as well [28, 31–33]m which led the WHO in 2021 to recommend Xpert-Ultra in several pulmonary and extrapulmonary samples, including stool but not urine samples [2].

In South Sudan, tuberculosis remains a major public health concern, with an estimated tuberculosis incidence in 2021 of 227 per 100 000 population (18% of cases affecting children <15 years old) and an overall treatment coverage of 72% [34]. The HIV prevalence in the country was 2.5% with, antiretroviral coverage of 18% in 2020 [35]. Internally displaced people living in the camps have an increased risk of tuberculosis [36]; by the end of 2023, 67 000 internally displaced people lived in Malakal. Médecins Sans Frontières (MSF) started support to the Ministry of Health in Malakal (Upper Nile state) by the end of 2013 due to a conflict-related humanitarian crisis. MSF is currently supporting primary and secondary healthcare in Malakal Protection of Civilians site and Town, while supporting the rural area through a decentralized model of healthcare.

Guinea-Bissau is a high-burden country for HIV and tuberculosis. In 2021, the estimated tuberculosis incidence was 361 per 100 000 population (4% of cases affecting children, likely underestimated), with overall treatment coverage of 33% [37]. The HIV prevalence in the country was estimated at 5% in 2018, with antiretroviral coverage of 34%, albeit 8% in pediatric HIV [38]. The rate of global acute malnutrition is estimated at 5% in children [39]. MSF supported the pediatric emergency and intensive care unit at Simão Mendes Hospital in Bissau from 2017 to 2020. The current study evaluates the performance of Xpert-Ultra with stool and urine samples and its additional diagnostic yield in a cohort of children ≤15 years of age (and subpopulations) with presumptive tuberculosis in 2 sub-Saharan hospital contexts.

## METHODS

### Study Design

This multicentric cross-sectional study took place at Malakal Teaching Hospital and Protection of Civilians hospitals from November 2019 to June 2023 and at Simão Mendes National Hospital between July 2019 and April 2020. Children with presumptive tuberculosis underwent clinical and laboratory evaluation.

### Study Population, Clinical, and Laboratory Procedures

Children aged 6 months to 15 years were considered presumptive tuberculosis case patients if they had persistent cough for >2 weeks, unexplained fever for >1 week, or signs of extrapulmonary tuberculosis, such as gibbous deformity of the spine, lymphadenopathy, subacute meningitis, distended abdomen with ascites, diarrhea for >2 weeks, painless enlarged joints, or pleural effusion.

Presumptive tuberculosis cases were also identified after a week of inpatient admission, characterized by low weight gain despite nutritional treatment, persistent pneumonia or cough despite adequate antibiotic therapy, persistent fever (>38°C), and persistent or aggravated fatigue. Patients were screened for tuberculosis based on their medical history and clinical presentation, including tuberculosis contacts, past tuberculosis treatment, or HIV infection. Physical examination was conducted in all patients and included relevant anthropometric measures (see “Data Collection and Analysis”). All patients with unknown HIV status were tested for HIV, and all children were tested with Xpert-Ultra for ≥1 pulmonary or extrapulmonary sample (considered the reference standard), 1 stool sample, and 1 urine sample.

Respiratory samples included nasopharyngeal aspirate, gastric lavage, and spontaneous sputum samples; sample collection procedures are described in Supplementary File 1. Extrapulmonary samples included lymph node puncture, pus aspirate, and ascitic, pleural, and cerebrospinal fluid samples. Stool and urine samples were collected from all patients as well. Standard operating procedures for Xpert-Ultra analysis of stool and urine samples can be found in Supplementary File 2. All specimens were kept between 2°C and 8°C until processing, which was done within 24 hours.

All patients underwent diagnostic evaluation and were included in 1 of 3 categories: confirmed tuberculosis (patients with ≥1 Xpert-Ultra–positive sample), unconfirmed tuberculosis (clinical tuberculosis diagnosis without Xpert-Ultra confirmation according to a clinical decision–based algorithm, which can be found in Supplementary File 3), and unlikely tuberculosis (Xpert-Ultra results negative, alternative diagnosis with adequate response to alternative treatments, and tuberculosis treatment not started). Patients with tuberculosis (confirmed or unconfirmed) were categorized according to the clinical presentation as pulmonary, extrapulmonary, or disseminated tuberculosis (when tuberculosis affected >1 site).

Xpert-Ultra was used to determine the specimens as follows: *Mycobacterium tuberculosis* not detected (or negative) or *M tuberculosis* detected (positive or trace); specimens with *M tuberculosis* detected were categorized as rifampicin resistance detected, not detected, or indeterminate. Any indeterminate or invalid result entailed repeating the test, and only considered invalid when the second test had the same result.

In Malakal, MSF supported the tuberculosis program in collaboration with the Ministry of Health all along the study. In Bissau, all results were communicated to the Ministry of Health, which was responsible for treatment initiation.

### Data Collection and Analysis

All data were collected in an anonymized, structured, paper-based forms, and managed using Research Electronic Data Capture (REDCap) software. Data were analyzed using SPSS (version 21; IBM) and R (version 4.3.1) software.

Continuous variables were reported using means and standard deviations (SD) or medians and interquartile ranges as appropriate and expressed as ordinal categories with frequencies. Frequencies were presented with corresponding 95% confidence intervals (CIs). Age-appropriate anthropometric indicators were calculated using the WHO Multicentre Growth Reference. Severe acute malnutrition (SAM) was defined based on WHO criteria (weight-for-height *z* score less than −3 SDs, or mid–upper arm circumference <11.5 cm, or presence of bilateral edema for children <5 years old and body mass index–for-age *z* score less than −3 SDs for those aged 5–15 years) [40].

The diagnostic accuracy of Xpert-Ultra in stool and urine samples for tuberculosis detection was validated against Xpert-Ultra in pulmonary and/or extrapulmonary samples, which were considered the reference standard for our study. Diagnostic accuracy was calculated as sensitivity, specificity, positive predictive value, and negative predictive value. All measures of accuracy were expressed as percentages with 95% CIs. Diagnostic yield was defined as the proportion of tuberculosis-positive results obtained from the test, regardless of whether those results were true-positives or false-positives.

Statistical differences were tested in univariable analyses using χ^2^, Fisher exact, or Kruskal-Wallis rank sum tests, as appropriate. All estimates are presented with their respective 95% CIs. For statistical comparisons on secondary objectives, differences were considered statistically significant at *P* < .05.

### Ethics and Patient Consent Statement

Ethical approval was obtained from the MSF Ethics Review Board (identifier [ID] 18116), the Guinea-Bissau National Health Ethics Committee (ID 009/CNES/INASA/2019), and the South Sudan Ministry of Health Ethical Review Board (ID MOH/ERB 20/2019). Written informed consent was received from a legal guardian and further verbal assent from children aged ≥10 years.

## RESULTS

### Demographics and Clinical Presentation

**Figure 1.**
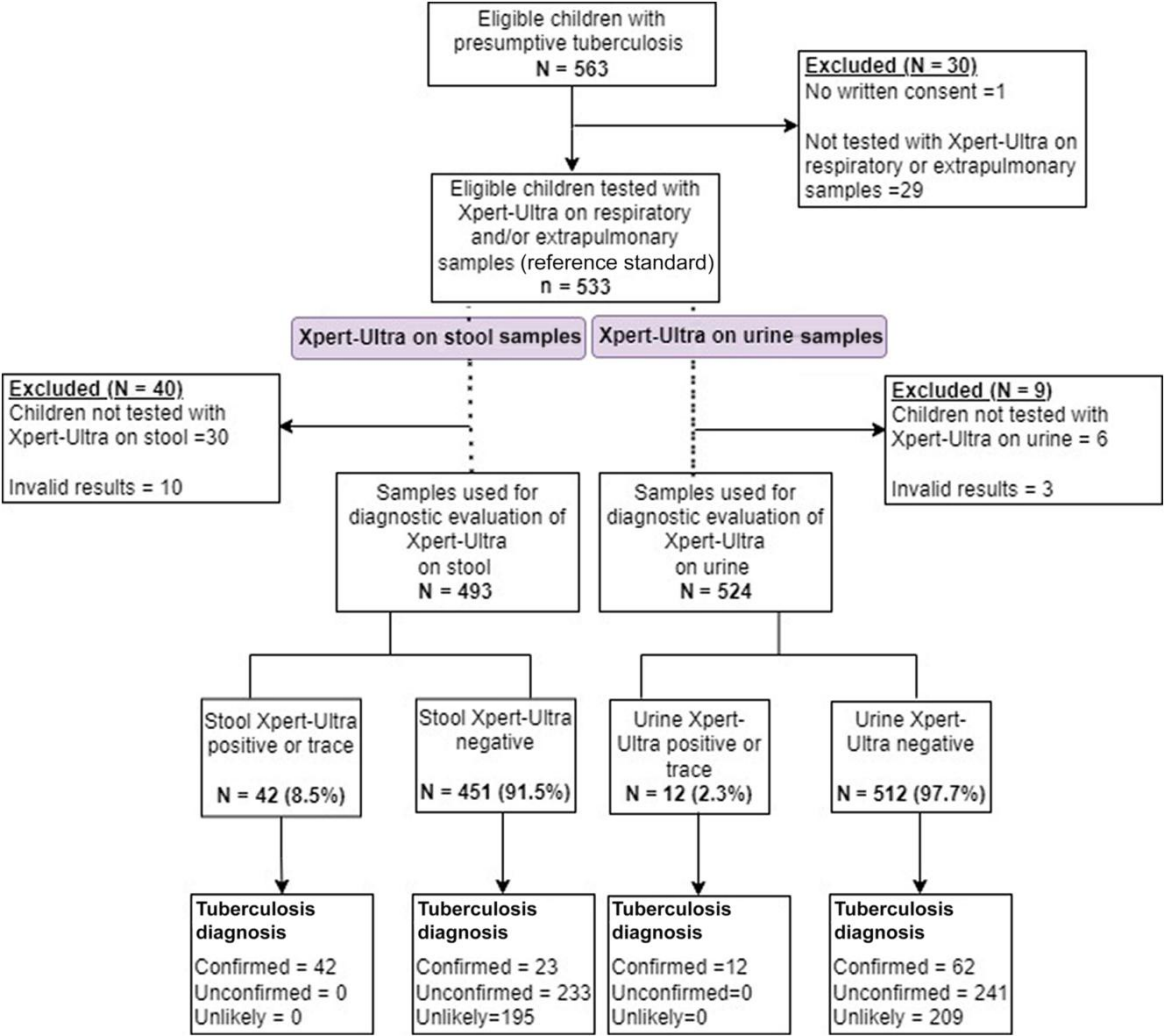
Flowchart of participants through the study.

A total of 563 patients were enrolled between July 2019 and April 2020 in Guinea-Bissau and from November 2019 to June 2023 in South Sudan. Thirty patients were excluded from the analysis due to absence of written consent (n = 1) or absence of a reference-standard sample (n = 29). For the analysis of Xpert-Ultra with stool samples, 40 additional patients were excluded due to absence of sample or invalid results; thus, a total of 493 patients were included in the subanalysis. For the Xpert-Ultra in urine analysis, 9 patients were excluded due to absence of sample or invalid result, with a total of 524 children included in the subanalysis. Figure 1 shows the flowchart of the participants in the study.

Demographic and clinical study characteristics of the participants are shown in Table 1. Of 533 patients with presumptive tuberculosis, 323 (61%) had a final diagnosis of tuberculosis, 75 (14.1%) presented with Xpert-Ultra confirmed tuberculosis and, among them, only 1 of 75 (1.3%) had rifampicin-resistant tuberculosis. Two hundred forty-eight patients (46.5%) had unconfirmed tuberculosis, and 210 (39.3%) had unlikely tuberculosis. A total of 93 patients (17.4%) presented with HIV infection, and 331 (62.1%) with SAM.

**Table 1.** Baseline Demographic and Clinical Characteristics of Children With Presumptive Tuberculosis at Simão Mendes Hospital and Malakal Project

Characteristic	Children, No. (%)^a^					*P* Value
	Overall (N = 533)	Confirmed Tuberculosis (n = 75)	Unconfirmed Tuberculosis (n = 248)	Total Tuberculosis (n = 323)	Unlikely Tuberculosis (n = 210)	
Age group						
0 to <2 y	166 (31)	15 (20)	92 (37)	107 (33.1)	59 (28)	.3
2 to <5 y	116 (22)	23 (31)	50 (20)	73 (22.6)	43 (20)	
5–15	251 (47)	37 (49)	106 (43)	143 (44.3)	108 (51)	
Age, median (IQR), mo	48 (18–108)	48 (24–99)	54 (32–108)	36 (16–96)	60 (20–120)	.05
Female sex	272 (51)	36 (48)	130 (53)	166 (52)	106 (50)	.8
Data missing	2 (0.4)	2 (1.5)	0 (0)	…	…	…
Tuberculosis history present	30 (5.6)	3 (4.0)	17 (6.9)	20 (6.2)	10 (4.8)	.8
Data missing	3 (0.6)	0 (0)	2 (0.8)	2 (0.6)	1 (0.5)	…
Tuberculosis contact present	168 (32)	32 (43)	84 (34)	116 (36)	52 (25)	.02^b^
Unknown	11 (2.1)	3 (4.0)	3 (1.2)	6 (2)	5 (2.4)	…
Children with SAM	331 (62)^c^	41 (55)	167 (68)	208 (65)	123 (59)	.17
Data missing	3 (0.6)	0 (0)	2 (0.8)	2 (0.6)	1 (0.5)	…
Children with HIV	93 (17)^d^	10 (14)	61 (25)	71 (22)	22 (10)	<.001^b^
Data missing	1 (0.2)	1 (1.3)	0 (0)	1 (0.3)	0 ()	…
CD4 cell count <200/μL^e^	26 (34)	4 (44)	15 (29)	19 (32)	7 (41)	.46
Data missing	17 (18.3)	2 (20)	10 (16.4)	12 (16.9)	5 (22.7)	…
On ART^e^	40 (43)	7 (64)	23 (38)	30 (42)	10 (45)	.9
Unknown	1 (1.1%)	1 (9.1%)	0	1 (1.4)	0	…
Tuberculosis type^f^						
Disseminated	92 (28.5)	33 (44)	59 (24)	92 (28.4)	…	-^g^
Extrapulmonary tuberculosis	70 (21.7)	19 (25)	51 (21)	70 (21.7)	…	
Pulmonary tuberculosis	161 (49.8)	23 (31)	138 (56)	161 (49.8)	…	
Cough	399 (75)	54 (72)	190 (77)	244 (76)	155 (74)	.7
Tachypnea	19 (3.6)	5 (6.7)	9 (3.6)	14 (4.3)	5 (2.4)	.2
Hypoxemia (SpO_2_ <92%)	50 (9.4)	6 (8.0)	34 (14)	40 (12)	10 (4.8)	.003^b^
Fever	440 (83)	60 (80)	201 (81)	261 (81)	179 (85)	.2
Weight loss	374 (70)	45 (60)	181 (73)	226 (71.3)	148 (71)	.4
Data missing	2 (0.4)	0 (0)	1 (0.4)	1 (1.4)	1 (0.5)	…
Gibbous	28 (5.3)	9 (12)	15 (6.0)	24 (7.4)	4 (1.9)	.005^b^
Lymph nodes	64 (12)	21 (28)	29 (12)	50 (15)	14 (6.7)	.002^b^
Subacute meningitis	5 (0.9)	0 (0)	3 (1.2)	3 (0.9)	2 (1.0)	>.9
Abdomen distended	28 (5.3)	3 (4.0)	15 (6.0)	18 (5.6)	10 (4.8)	.7
Diarrhea	49 (9.2)	3 (4.0)	27 (11)	30 (9.3)	19 (9.0)	>.9
Painless enlarged joints	8 (1.5)	1 (1.3)	5 (2.0)	6 (1.9)	2 (1.0)	.5
Pleural effusion	7 (1.3)	1 (1.3)	6 (2.4)	7 (2.2)	0 (0)	.046^b^
Other extrapulmonary tuberculosis signs	6 (1.1)	2 (2.7)	3 (1.2)	5 (1.5)	1 (0.5)	.4
No signs of extrapulmonary tuberculosis	345 (65)	36 (48)	150 (60)	186 (58)	159 (76)	<.001^b^

Abbreviations: ART, antiretroviral therapy; HIV, human immunodeficiency virus; IQR, interquartile range; SAM, severe acute malnutrition; SpO_2_, oxygen saturation measured by pulse oximetry.

^a^Data represent no. (%) of children unless otherwise specified.

^b^Significant at *P* < .05

^c^Of 331 children with SAM, 70 (21.1%) were living with HIV.

^d^Of 93 children with HIV, 70 (75.3%) had SAM.

^e^Excluding data from children without HIV.

^f^Excluding data from children with unlikely tuberculosis.

^g^Unlikely TB.

Comparative data between Malakal and Bissau can be found in Supplementary Table 1. Comparing Malakal (n = 400) and Bissau (n = 133) cohorts, patients from Bissau were older, had more history of tuberculosis contact, and presented with more HIV infection and lower CD4 cell counts. Clinically, children in Malakal presented with greater weight loss, fever, diarrhea, and prevalence of extrapulmonary tuberculosis.

In the total cohort, the median age (interquartile range) was 48 (18–108) months and 282 patients (52.9%) were <5 years old. Demographic characteristics (including age, sex, and previous history) were similar in all tuberculosis groups. Comparing patients with tuberculosis with unlikely tuberculosis patients, those with tuberculosis presented with a similar proportion of SAM, but those with tuberculosis had more HIV infection and lower CD4 cell counts and more exposure to tuberculosis. In terms of clinical presentation, hypoxemia, gibbous, peripheral lymph nodes, and pleural effusion were significantly more common in patients with tuberculosis.

### Performance of Xpert-Ultra in Stool and Urine Samples

The performance of Xpert-Ultra in stool samples is described in Table 2. The overall diagnostic yield in stool samples was 8.5% (42 of 493), with a third of positives by trace result (14 of 42). Overall, the sensitivity of Xpert-Ultra in stool samples was 62.5% (95% CI, 49.4%–74%), and the specificity was 98.4% (96.7%–99.2%); breakdowns by HIV, nutrition, and age subpopulation are also shown in the Table 2. The diagnostic accuracy was 94.3% (95% CI, 91.9%–96.0%), and the Cohen κ value was 0.68 (.60–.77).

**Table 2.** Diagnostic Accuracy of Xpert MTB/RIF Ultra Assay in Stool and Urine Samples

Sample Type Assayed	Samples, No. (%)					Value, % (95% CI)			
	Total	TP	FP	FN	TN	Sensitivity	Specificity	PPV	NPV
Stool samples									
All children	493	35	7	21	430	62.5 (49.4–74)	98.4 (96.7–99.2)	83.3 (69.4–91.7)	95.3 (93–96.9)
Children with HIV	86	7	0	1	78	87.5 (52.9–97.8)	100 (95.3–100)	100 (64.6–100)	98.7 (93.2–99.8)
Children <5 y old	262	16	4	11	231	59.3 (40.7–75.5)	98.3 (95.7–99.3)	80 (58.4–91.9)	95.5 (92.1–97.4)
Children with SAM	308	23	3	9	273	71.9 (54.6–84.4)	98.9 (96.9–99.6)	88.5 (71–96)	96.8 (94.1–98.3)
Validated against pulmonary samples^a^	470	34	6	15	415	69.4 (55.5–80.5)	98.6 (96.9–99.4)	85 (70.9–92.9)	96.5 (94.3–97.9)
Validated against extrapulmonary samples^a^	50	6	1	12	31	33.3 (16.3–56.3)	96.9 (84.3–99.5)	85.7 (48.7–97.4)	72.1 (57.3–83.3)
Urine samples (all children)	524	7	3	58	456	13.9 (7.5–24.3)	99.4 (98.1–99.8)	75 (46.8–91.1)	89.1 (86.1–91.5)

Abbreviations: CI, confidence interval; FN, false-negative; FP, false-positive; HIV, human immunodeficiency virus; NPV, negative predictive value; PPV, positive predictive value, SAM, severe acute malnutrition; TN, true-negative; TP, true-positive.

^a^In 23 children, both pulmonary and extrapulmonary samples were obtained.

The performance of Xpert-Ultra in urine samples is also described in Table 2. The overall diagnostic yield of Xpert-Ultra in urine samples was 2.3% (12 of 524), with 25.0% (3 of 12) of positives by trace result. Overall, the sensitivity (95% CI) of Xpert-Ultra in urine samples was 13.9% (7.5%–24.3%), and the specificity was 99.4% (98.1%–99.8%). Further analysis on subpopulations was not carried out due to the small number of Xpert-Ultra–positive urine samples. The diagnostic accuracy of Xpert-Ultra in urine samples was 89.1% (95% CI, 85.8%–91.2%), and the Cohen κ was 0.20 (0.14–0.26). Among these 12 children with Xpert-Ultra–positive urine samples, 7 (58.3%) presented with disseminated tuberculosis.

In a total of 9 patients (12.0% [9 of 75]), tuberculosis was diagnosed based on a positive Xpert-Ultra result in a stool and/or urine sample alone, as the reference-standard sample was negative. In Supplementary Figure 1, the Venn diagram shows the concordance by sample type and includes only patients who had reference standard, stool, and urine specimens with a positive result (n = 490). Supplementary Table 2 provides descriptive details for patients who presented with Xpert-Ultra results only for stool and/or urine samples.

### Diagnostic Yield by Pulmonary or Extrapulmonary Samples and Combination of Tests/Samples

The positivity rate per sample is shown in Supplementary Table 3. For pulmonary samples, the overall positivity rate was 9.8% (49 of 500), with a trace result in 10.2% (5 of 49). The positivity rates for nasopharyngeal aspirate and gastric lavage samples were similar (9.5% and 9.2%, respectively). Extrapulmonary samples had a positivity rate of 32.0% (16 of 50), with the highest rates found in lymph node samples (35.0%) and pus or tissue samples (33.3%). Among extrapulmonary samples, 19.0% (4 of 21) had a trace result.

The diagnostic yields of different combinations samples are shown in Table 3, which excludes samples from patients without Xpert-Ultra performed in stool samples. For patients with pulmonary tuberculosis, the most efficient combination was sputum and stool samples (24.3%), which can be obtained only in older children; for younger children, gastric aspirate and stool samples had a diagnostic yield of 16.4% in patients with tuberculosis, but Xpert-Ultra in stool samples did not increase the diagnostic yield of nasopharyngeal aspirates (13.5%). The diagnostic yield of nasopharyngeal aspirates was higher in patients with extrapulmonary tuberculosis, namely, 41.2% when combined with stool samples, with the highest increase in yield found for cervical lymph node tuberculosis (6.2%, from 35.0% to 41.2%).

**Table 3.** Diagnostic Yields of Different Test/Combinations

Samples Tested With Xpert-Ultra Assay	Diagnostic Yield, % (No./Total)	
	All Patients	Patients With Tuberculosis
Respiratory only	9.4 (44/470)	15.7 (44/281)
Respiratory + stool	10.9 (51/470)	18.1 (51/281)
Sputum	12.0 (15/125)	21.4 (15/70)
Sputum + stool	13.6 (17/125)	24.3 (17/70)
Gastric aspirate	8.5 (24/283)	14.0 (24/171)
Gastric aspirate + stool	9.9 (28/283)	16.4 (28/171)
Nasopharyngeal aspirate	8.6 (5/58)	13.5 (5/37)
Nasopharyngeal aspirate + stool	8.6 (5/58)	13.5 (5/37)
Extrapulmonary only	32.0 (16/50)	37.2 (16/43)
Extrapulmonary + stool	36.0 (18/50)	41.9 (18/43)
Extrapulmonary + urine	33.9 (20/59)	40.8 (20/49)
Lymph node	35.0 (14/40)	41.2 (14/34)
Lymph node + stool	40.0 (16/40)	47.1 (16/34)
Pus or tissue	33.3 (2/6)	40.0 (2/5)
Pus or tissue + stool	33.3 (2/6)	40.0 (2/5)
Stool only	8.5 (42/493)	14.1 (42/298)
Urine only	2.3 (12/524)	3.8 (12/315)
Stool and urine only	9.2 (45/490)	15.3 (45/295)

### Xpert-Ultra Diagnostic Yield by HIV and Nutritional Status and Age

The diagnostic yields of respiratory and extrapulmonary samples were similar by age and by HIV and nutritional status and is presented in Table 4. The diagnostic yields in stool and urine samples were similar per category.

**Table 4.** Diagnostic Yield of Xpert-Ultra by Sample Type and by Patient Age, Human Immunodeficiency Virus, and Nutritional Status

Patient Status by Sample Type	Overall	Tuberculosis	No Tuberculosis	OR (95% CI)	*P* Value
Respiratory samples					
<5 y old	268 (53.5)	24 (49)	244 (54)	0.8 (.5–1.5)	.51
≥5 y old	233 (46.5)	25 (51)	208 (46)	…	
SAM	321 (64.3)	32 (65.3)	289 (64.2)	1.1 (.6–2.0)	.88
No SAM	178 (35.7)	17 (34.7)	161 (35.8)	…	
HIV	89 (17.8)	9 (18.8)	80 (17.7)	1.1 (.5–2.3)	.86
No HIV	411 (82.2)	39 (81.3)	372 (82.3)	…	
Extrapulmonary samples					
<5 y old	30 (50)	9 (42.9)	21 (53.8)	0.6 (.2–1.9)	.42
≥5 y old	30 (50)	12 (57.1)	18 (46.2)	…	
SAM	23 (39)	8 (38.1)	15 (39.5)	0.9 (.3–2.8)	.92
No SAM	36 (61)	13 (61.9)	23 (60.5)	…	
HIV	5 (8.3)	1 (4.8)	4 (10.3)	0.4 (.01–4.9)	.84
No HIV	55 (91.7)	20 (95.2)	35 (89.7)	…	
Stool samples					
<5 y old	262 (53.1)	20 (47.6)	242 (53.7)	0.8 (.4–1.5)	.45
≥5 y old	231 (46.9)	22 (52.4)	209 (46.3)	…	
SAM	308 (62.7)	26 (61.9)	282 (62.8)	1.0 (.5–1.9)	.91
No SAM	183 (37.3)	16 (38.1)	167 (37.2)	…	
HIV	86 (17.5)	7 (17.1)	79 (17.5)	1.0 (.4–2.3)	.94
No HIV	406 (82.5)	34 (82.9)	372 (82.5)	…	
Urine samples					
<5 y old	274 (52.3)	7 (58.3)	267 (52.1)	1.3 (.4–4.1)	.67
≥5 y old	250 (47.7)	5 (41.7)	245 (47.9)	…	
SAM	322 (61.8)	11 (91.7)	311 (61.1)	7.0 (1.0–302.9)	.05
No SAM	199 (38.2)	1 (8.3)	198 (38.9)	…	
HIV	91 (17.4)	3 (25)	88 (17.2)	1.6 (.3–6.6)	.7
No HIV	432 (82.6)	9 (75)	423 (82.8)	…	

Abbreviations: CI, confidence interval; HIV, human immunodeficiency virus; OR, odds ratio; SAM, severe acute malnutrition.

## DISCUSSION

To our knowledge, this is the first study to investigate the diagnostic accuracy of Xpert-Ultra in urine samples for the diagnosis of tuberculosis in children and the first to assess Xpert-Ultra in stool and urine samples for pediatric extrapulmonary tuberculosis and in HIV subpopulations. In addition, this study provides relevant results on the diagnostic utility of Xpert-Ultra in stool and urine samples in field conditions within resource-limited settings and in a large cohort of children with presumptive tuberculosis (n = 533) and with a final tuberculosis diagnosis (n = 323). It is also the first peer-reviewed study to report tuberculosis diagnosis in a pediatric population in South Sudan.

This multicentric study was conducted in 2 high-burden HIV, tuberculosis, and malnutrition settings in Guinea-Bissau and South Sudan. Even though cohorts presented with significant differences in some relevant demographics and in clinical presentations, such as tuberculosis contact or HIV coinfection, we found similar proportions of final tuberculosis diagnoses (59.3%–64.7%) among children with presumptive tuberculosis across sites. These were higher than those reported in other studies [31–33], a difference likely attributed to the high tuberculosis incidence in the context of an effective clinical decision diagnostic algorithm. Microbiological confirmation of tuberculosis was achieved in 24.9%–30.2% of tuberculosis cases, which is in line with WHO observations [3], and only 1.3% (1 of 75 confirmed patients) presented with rifampicin-resistant tuberculosis.

In our study, we found that a third of patients with confirmed extrapulmonary tuberculosis had positive Xpert-Ultra results with stool samples, which has not been reported in other studies. Of note, patients with cervical lymph node tuberculosis experienced an increase of 6.2% in diagnostic yield when combined with stool samples. This may indicate either nonsymptomatic concomitant pulmonary tuberculosis or a gastrointestinal infection and would support recommending Xpert-Ultra testing in stool samples for all presumptive pediatric tuberculosis cases, not only pulmonary tuberculosis cases.

In terms of test performance, the sensitivity of Xpert-Ultra in urine samples was low, and its use would not be systematically recommended from a programmatic perspective at this stage, considering the low number of patients with Xpert-Ultra–positive urine samples (n = 12). Of note, 58.3% (7 of 12) presented with disseminated tuberculosis, though only one-quarter (3 of 12) were living with HIV; Xpert-Ultra in urine samples has shown some utility in disseminated tuberculosis but has mainly been studied in people with advanced HIV [26, 41]. We found a nonsignificant increase in diagnostic yield in children with SAM compared with those without SAM, suggesting that further studies may help identify and confirm whether Xpert-Ultra in urine samples could play a useful role in specific subpopulations.

Xpert-Ultra in stool samples showed a sensitivity of 62.5% overall, and 69.4% among pulmonary tuberculosis cases, as well as a diagnostic accuracy of 94.3%. These were higher than reported in previous studies that evaluated the performance of Xpert-Ultra with stool samples in pulmonary tuberculosis cases with tuberculosis culture as the reference standard, where sensitivities ranged between 45.5% and 60.3% [31–33]. The standard operating procedure for stool processing was based on the MSF standard operating procedures (2018; Supplementary File 2). Compared with WHO-recommended stool processing methods [42], centrifugation was used, which may explain the higher sensitivity compared with other methods that did not use centrifugation and for which sensitivity ranged between 46.8% and 52.1% [42–44].

We also noted an additional yield of 10.7% (8 of 75 patients) for tuberculosis diagnoses based on Xpert-Ultra findings in stool samples. This suggests that screening children with presumptive tuberculosis with Xpert-Ultra in stool samples could in some cases shorten the time to tuberculosis diagnosis. Those meeting the criteria and having a positive Xpert-Ultra result with stool samples could commence treatment. In case of a negative result, a second test in a respiratory or extrapulmonary specimen could help confirm tuberculosis disease. Of note, this may imply an increase in consumables and, consequently, in cost. Further studies on cost-benefit may be considered.

In patients with a final diagnosis of tuberculosis, the diagnostic yields of Xpert-Ultra in stool and urine samples were 14.1% and 3.8%, respectively. Compared with the cohort of presumptive tuberculosis cases reported by Kabir et al [31], the diagnostic yield of Xpert-Ultra in stool samples was similar. In pulmonary tuberculosis cases, sputum samples had the higher diagnostic yield, with 21.4%, but these samples can be obtained only in older children capable of expectorating. Induced sputum samples could be an alternative, but they was not available in our setting. For younger children, gastric aspirates in combination with stool samples presented with a diagnostic yield of 16.4%. Despite the latest WHO recommendation, we did not find a significant increase when using stool samples in combination with nasopharyngeal aspirate (13.5%), albeit with a low number of confirmed cases (n = 5).

The WHO recommends Xpert-Ultra as a first test for children with presumptive tuberculosis, despite a lower specificity [28] due to its increased sensitivity compared with conventional cartridge tests; the effort to increase tuberculosis diagnosis in children outweighs the risk of overtreating patients [11]. It is worth noting that a high proportion of patients with confirmed tuberculosis presented with a trace result in Xpert-Ultra: 10.2% in pulmonary, 19.0% in extrapulmonary, 33.3% in stool, and 25.0% in urine samples, congruent with other studies [31]. Those results were interpreted as true-positives, considering the clinical condition of our pediatric cohort in field conditions with high HIV/SAM prevalence; Xpert-Ultra was consequently not repeated. Of note, a small proportion of invalid results had to be excluded from the analysis despite repeating the test (2.4% [13 of 533]).

When examining HIV, SAM, and <5-year-old subpopulations, we found a nonsignificant trend suggesting greater sensitivity of Xpert-Ultra with stool samples in children with HIV (87.5%) or SAM (71.9%), without a substantial drop in specificity, similar to the findings described by Kabir et al [31], who found a sensitivity of 71% children with SAM; unpublished data available in a Cochrane review report a sensitivity of 50% in children living with HIV [28]. If confirmed in larger samples, this would support the systematic use of Xpert-Ultra with stool samples in those key vulnerable populations. The diagnostic yields were similar in each subgroup in respiratory and extrapulmonary samples.

The main limitation of our study was the impossibility to access tuberculosis culture, in addition to having an imperfect reference standard for unconfirmed tuberculosis cases, which is a limitation for the calculation of specificity. The study was conducted in 2 settings with high tuberculosis incidence and HIV/malnutrition prevalence, which may compromise the generalizability of our results to lower prevalence settings. In addition, trace results were not repeated, which may ultimately overestimate sensitivity and underestimate specificity, and Xpert-Ultra has a lower specificity compared with conventional cartridge tests.

In conclusion, this study was carried out during routine care provision in 2 resource-limited and high-tuberculosis-burden contexts and found that Xpert-Ultra in stool samples showed moderate to high sensitivity and high specificity compared with the reference standard and an added diagnostic yield when the reference-standard results were negative. Xpert-Ultra in stool samples was also useful for extrapulmonary presentations. The test performance of Xpert-Ultra in urine samples was low.

## Supplementary Material

ofae221_Supplementary_Data

## Supplementary Data

Supplementary materials are available at *Open Forum Infectious Diseases* online. Consisting of data provided by the authors to benefit the reader, the posted materials are not copyedited and are the sole responsibility of the authors, so questions or comments should be addressed to the corresponding author.

## Notes

***Author contributions***. Conception or design of the work: L. M. P. Data collection: J. G., E. B., and R. G., and L. F. Data analysis: R. M. Interpretation of data analysis: L. M. P. and R. M. Drafting the manuscript: L. M. P. Critical revision of the manuscript: L. M. P., R. M., M. J. S., I. M., and A. E. L. All authors contributed substantially to designing, performing, or reporting the study, and all authors read and approved the final manuscript.

***Data sharing statement***. Data are available to be shared on request, per MSF data sharing policies.

***Financial support*.** This work was supported by Médecins Sans Frontières Operational Centre Barcelona-Athens (Spain).
