# Supplementary material for: Xpert-Ultra Assay in Stool and Urine Samples to Improve Tuberculosis Diagnosis in Children: The Médecins Sans Frontières Experience in Guinea-Bissau and South Sudan
Source: Open Forum Infect Dis. 2024 May 2;11(5):ofae221. doi: 10.1093/ofid/ofae221 (PMC11119760; doi:10.1093/ofid/ofae221)
Supplement: ofae221_Supplementary_Data [file ofae221_supplementary_data.zip › OFID_Supplementary material_Xpert-Ultra stool_urine_050424.docx]

**Supplementary file 1: Standard operating procedures for sample collection of naso-pharyngeal aspirate and gastric lavage**

1. **SOP for Naso-pharyngeal aspirate (NPA)**

Equipment required:

- Disposable gloves and respirator masks (N95)
- Suction apparatus
- Sterile 6/7/8 G mucus extractor or nasogastric catheter
- Normal saline (0.9%) 5 ml
- Sterile syringe 50 ml (single use)

Performing nasopharyngeal aspiration:

1. Infection control measures: All personnel responsible for collecting respiratory specimens must wear personal protective equipment. The procedure will take place in a dedicated cough room or should be fitted with a door that can lock and windows that open to the outside. Alternatively, samples may be collected outside in the open air or in sputum collection booth/other appropriate structure housed outdoors.

2. The child’s nose is cleaned with saline drops. If old enough, the child can be asked to blow the nose into a tissue. If the nasal mucus is too thick to be removed with the measures above, it can be suctioned prior to nasopharyngeal aspiration. A soft catheter size F6/7 is used for suctioning and is discarded immediately afterwards.

4. Two drops of sterile saline are instilled into each nostril.

5. The length of the cannula used for aspirating the NPA sample is measured as the distance from nostril to tragus of the ear; then the posterior nasopharynx is suctioned using a soft plastic cannula connected to a mucus trap.

6. Suctioning is activated only when the tip of the cannula is in the posterior nasopharynx. When the cannula is passed through the nostrils (during introduction and extraction), the suction is de-activated.

7. Transfer full volume of sample into a sterile container (Falcon tube).

8. Clean Falcon tube with alcohol swabs.

9. Label sample: sample type and number, date, time, total sample volume.

10. Place specimen in sample bag, seal and put into a cold box for transport to lab.

1. **SOP for Gastric lavage**

Equipment required:

- Gloves and respirator
- Suction catheter (6, 7, 8F)
- Sputum container
- 50 ml syringe
- Sterile water

Performing gastric lavage:

1. Infection control measures: All personnel responsible for collecting respiratory specimens must wear personal protective equipment. The procedure will take place in a dedicated cough room or should be fitted with a door that can lock and windows that open to the outside. Alternatively, samples may be collected outside in the open air or in sputum collection booth/other appropriate structure housed outdoors.

2. Child needs to be fasting for at least 4 hours and the procedure will take place early in the morning.

3. Place the child in a half-sitting or sitting position in the adult’s arms.

4. Insert a nasogastric tube and check that it is correctly placed.

5. First suction to collect the gastric fluid and place it in the sputum container, then rinse the stomach with 30 ml of sterile water and suction again. Add the suctioned fluid to the first sample.

6. Start culture within 4 hours of collecting the sample. If there will be more than four hours’ delay, neutralize with 100 mg of sodium bicarbonate.

**Supplementary file 2: Standard operating procedures for Xpert-Ultra on stool and urine**

1. **SOP XPERT-ULTRA ON STOOL**

**Materials**

- Xpert DX System
- Xpert Instrument, computer, barcode reader
- Xpert-Ultra cartridge and sample reagent bottle
- Materials required bud not provided in the kit
- Centrifuge
- Gloves
- Pipette (capable of dispensing 2mL)
- Biohazard disposable bags

**Safety, Health & Environment**

Treat all stool specimens as potentially infectious and follow basic universal precautions. Wear protective clothing (coat/apron and gloves) when handling the specimens.

**Specimen collection and storage**

Collect stool samples from children in a clean wide-open container. Stool samples must be processed within the day of collection. Fresh stool samples can be used within 3 hours if kept at room temperature.

**Reagent storage and preparation**

Xpert-Ultra cartridges must be stored at 2-28ºC. Do not use beyond expiration date and do not open the cartridge until you are ready to perform the test (use the cartridge within 30minutes after opening its lid).

**Test Procedure**

- Add an aliquot of 2-3 g (pea size) of stool sample into a centrifuge tube (with a lid) using a sterile disposable plastic loop
- Add 5 ml of Phosphate Buffered Saline (PBS) and vortex to homogenize the mixture
- Centrifuge the mixture at 3200x g for 15 min
- Add 2 ml of the Xpert reagent into 1ml of the re-suspended pellet mixture and mix thoroughly.
- Using the sterile pipette provided, aspirate the liquefied sample into the pipette until the meniscus is above the minimum mark. Add the mixture into the Xpert-Ultra cartridge and insert the cartridge into the Xpert instrument and conduct the assay according to the manufacturer’s instructions (follow your laboratory Sop for operation of the Xpert Instrument).

**Interpretation of test results**:

- Interpret the results as you normally do with results from sputum samples processed in Xpert Instrument.
- The results are produced by the Xpert DX System from measured fluorescent signals and embedded calculation algorithms and will be displayed in the “View Results” window. Lower Ct values represent a higher starting concentration of DNA template; higher Ct values represent a lower concentration of DNA template.
- MTB Detected=> MTB target DNA is detected. The MTB result will be displayed as High, Medium, Low or Very Low depending on the Ct value of the MTB target present in the sample.
- RIF Resistance Detected=> will be displayed if the mutation in the rpoB gene has been detected. This is only displayed in MTB detected results.
- MTB Not Detected=>MTB target DNA is not detected

**Quality control testing**

Each Xpert test cartridge is a self-contained test device with an in-built control for each sample. Normally, no external controls are required. The internal controls enable the system to detect specific failure modes within each for each sample.

- Instrument system control: Check status-it checks the optics, temperature of the module and the mechanical integrity of each cartridge. If the system controls fail, an ERROR test result will be reported.
- Probe Check control (PCC): after sample preparation, bead reconstitution and tube filling (prior to thermal cycling), multiple fluorescent readings are taken at different temperatures and compared to default setting. PCC controls for:
  - Missing target specific reagent (TSR) and or enzyme reagent beads which contain all primers, probes and internal control template.
  - Incomplete reagent reconstitution
  - Incomplete reaction tube filling
  - Probe degradation
- If the PCC fails, an ERROR test result will be reported.
- Sample processing control (SPC) assesses the effectiveness of the sample processing steps, including and up-to reaction tube filling. SPC ensures that the sample was correctly added to the cartridge and detects degradation of the enzyme(s) or other components of the system. SPC does not compete with target DNA.
  - SPC must be Positive when target is Negative
  - SPC can be Positive or Negative when the target is Positive
  - SPC passes if it meets the validated acceptance criteria. E2097: too less sample volume added (<1mL), E2096: no sample added
- Internal Quantitative Standard High and Low (IQS-H and IQS-L): IQS-H and IQS-L are two dry bead armored RNAs nonspecific to HIV in the form of a dry bead that goes through the whole GX process. The IQS-H and IQS-L are standards calibrated against the WHO 3rd International Standard. They are used for quantification by using lot specific parameters for the calculation of HIV-1 RNA concentration in the sample. The IQS-H and IQS-L pass if they meet the validated acceptance criteria. They run internally with every cartridge and they control for reagent performance due to improper storage and they confirm that reaction components are set up correctly.
- External Controls: not available in the kit, but they can be used (positive and negative controls).

1. **SOP XPERT-ULTRA ON URINE**

**Materials**

- Xpert DX System
- Xpert Instrument, computer, barcode reader
- Xpert MTB/Rif cartridge and sample reagent bottle
- Materials required bud not provided in the kit
- Centrifuge
- Gloves
- Pipette (capable of dispensing 2mL)
- Biohazard disposable bags

**Safety, Health & Environment**

Treat all stool specimens as potentially infectious and follow basic universal precautions. Wear protective clothing (coat/apron and gloves) when handling the specimens.

**Specimen collection and storage**

Collect midstream urine in a fresh standard urine collection container. Fresh urine samples can be used within 3 hours if kept at room temperature.

Urine samples should be stored at 2-8°C if the test is to be run within 3 days of collection.

- If testing is delayed more than 3 days, the samples should be frozen (-20°C or colder). For frozen or refrigerated urine bring all samples to room temperature one hour prior to use. Frozen samples may contain aggregates.
- All thawed samples must be centrifuged at 3000 g for 15 minutes at room temperature Specimens that have been frozen and thawed more than 3 times cannot be used.

**Reagent storage and preparation**

Xpert-Ultra cartridges must be stored at 2-28oC. Do not use beyond expiration date and do not open the cartridge until you are ready to perform the test (use the cartridge within 30minutes after opening its lid).

**Test Procedure**

- The sample of urine, 4ml, is centrifuged at 3000g for 5 minutes.
- Decant the supernatant and re-suspend the pellet/sediment in 2mL of the Xpert sample reagent.
- Thoroughly mix the re-suspension mixture, and add 2ml of the mixture into the Xpert-Ultra cartridge.
- Insert the cartridge into the Xpert instrument and proceed with testing (follow your laboratory Sop for operation of the Xpert Instrument).

**Interpretation of test results**:

- Interpret the results as you normally do with results from sputum samples processed in Xpert Instrument.
- The results are produced by the Xpert DX System from measured fluorescent signals and embedded calculation algorithms and will be displayed in the “View Results” window. Lower Ct values represent a higher starting concentration of DNA template; higher Ct values represent a lower concentration of DNA template.
- MTB Detected=> MTB target DNA is detected. The MTB result will be displayed as High, Medium, Low or Very Low depending on the Ct value of the MTB target present in the sample.
- RIF Resistance Detected=> will be displayed if the mutation in the rpoB gene has been detected. This is only displayed in MTB detected results.
- MTB Not Detected=>MTB target DNA is not detected

**Quality control testing**

Each Xpert test cartridge is a self-contained test device with an in-built control for each sample. Normally, no external controls are required. The internal controls enable the system to detect specific failure modes within each for each sample.

- Instrument system control: Check status-it checks the optics, temperature of the module and the mechanical integrity of each cartridge. If the system controls fail, an ERROR test result will be reported.
- Probe Check control (PCC): after sample preparation, bead reconstitution and tube filling (prior to thermal cycling), multiple fluorescent readings are taken at different temperatures and compared to default setting. PCC controls for:
  - Missing target specific reagent (TSR) and or enzyme reagent beads which contain all primers, probes and internal control template.
  - Incomplete reagent reconstitution
  - Incomplete reaction tube filling
  - Probe degradation
- If the PCC fails, an ERROR test result will be reported.
- Sample processing control (SPC) assesses the effectiveness of the sample processing steps, including and up-to reaction tube filling. SPC ensures that the sample was correctly added to the cartridge and detects degradation of the enzyme(s) or other components of the system. SPC does not compete with target DNA.
  - SPC must be Positive when target is Negative
  - SPC can be Positive or Negative when the target is Positive
  - SPC passes if it meets the validated acceptance criteria. E2097: too less sample volume added (<1mL), E2096: no sample added
- Internal Quantitative Standard High and Low (IQS-H and IQS-L): IQS-H and IQS-L are two dry bead armored RNAs nonspecific to HIV in the form of a dry bead that goes through the whole GX process. The IQS-H and IQS-L are standards calibrated against the WHO 3rd International Standard. They are used for quantification by using lot specific parameters for the calculation of HIV-1 RNA concentration in the sample. The IQS-H and IQS-L pass if they meet the validated acceptance criteria. They run internally with every cartridge and they control for reagent performance due to improper storage and they confirm that reaction components are set up correctly.
- External Controls: not available in the kit, but they can be used (positive and negative controls).

**Supplementary file 3: Médecins sans Frontières Standard Operating Procedure (SOP) for the diagnosis of TB in children**

Cough > 2 weeks or fever^a^ > 1 week or suspicion of

Extra pulmonary TB^b^

Day 1

………………………………………………………………………………………………………………………………………………….

**Repeat if likely TB:**

**Xpert-Ultra respiratory or extrapulmonary sample**

**Xpert-Ultra on stools and urine**

**Xpert-Ultra respiratory or extrapulmonary sample**

**Xpert-Ultra on stools and urine**

Repeat comprehensive assessment^c^:

- Poor weight gain / poor appetite test

- Persistent cough

- Persistent fever

- Fatigue or lethargy

- CXR suggestive of TB findings

Repeat comprehensive assessment^c^ and other investigations after 1 week

Is the child still symptomatic?

Antibiotics^e^, nutritional treatment or other treatment according to clinical findings

Comprehensive assessment^c^

Day 7

Not obvious TB

Continue antibiotics^e^, nutritional treatment or other treatment according to clinical findings

NO

NO

YES

Day 10-12

Is the child HIV exposed / infected or is a contact of a TB case?

YES

SAM

Start TB treatment

If obvious TB^d^

Start TB treatment

If obvious TB^d^

If not obvious TB

Start TB treatment in particular if child is:

- HIV positive or

- < 3 years or

- SAM or

- TST +

Start TB treatment

TB Unlikely

One present

≥2 present

None present^f^

1. Axillar temperature > 38ºC
2. Enlarged lymph nodes, gibbous, > 2 weeks diarrhoea, meningitis, malnutrition not improving with proper nutritional treatment without other obvious causes (I e. visceral leishmaniasis)
3. Comprehensive assessment includes:
   1. Clinical assessment
   2. Growth assessment
   3. Bacteriological test (sputum collection, naso-pharyngeal aspirate, gastric lavage, lymph node aspiration):

- Xpert MTB/RIF testing (sputum, nasopharyngeal aspirate, gastric lavage, lymph node aspirate, CSF);
- Xpert MTB/RIF in stools and urine;
- TB culture and DST (only at baseline):
- Tb LAM for HIV+ chldren

For children:

- < 5 years old: do nasopharyngeal aspirate or gastric lavage;
- From 5 years old until child can expectorate: gastric lavage;
- For children who can expectorate: sputum.
  1. POCUS (only at baseline)
  2. HIV testing if not yet performed (Appendix 16)
  3. When relevant and available: X-ray (CXR, spine)

1. Xpert MTB/RIF positive, CXR showing suggestive lesions (e.g. hilar lymphadenopathy, upper lobe infiltrates, and miliary picture), gibbous.
2. Broad spectrum antibiotics:

- **If no signs of severity:**
  - First line: amoxicillin PO for 7 days (NO fluoroquinolones);
  - If a second course of antibiotics is needed: azithromycin PO for 5 days.
- **If signs of severity**:
  - Parenteral antibiotics (ceftriaxone ± cloxacillin if *S.aureus* is suspected);
  - If a second course of antibiotic is needed: azithromycin PO for 5 days;
  - In addition: **PCP treatment** should be given presumptively to all HIV-exposed or infected children < 1 year of age and any older child with sever immune suppression and not on CTX prophylaxis. For all other HIV-exposed or infected children, it should be considered if there is poor response to broad spectrum antibiotics after 48 h.

1. Clinical response to broad-spectrum antibiotic does not rule out TB. Continue follow up to see if symptoms re-occur.

**Supplementary table 1:** Baseline demographic and clinical of children with presumptive TB at Simão Mendes hospital and Malakal project

| Characteristic | Overall,  N = 533 | Bissau,  N = 133 | Malakal,  N = 400 | p-value*^2^* |
| --- | --- | --- | --- | --- |
| Age group (years) |  |  |  | **0.004** |
| 0-<2 | 166 (31%) | 34 (26%) | 132 (33%) |  |
| 2-<5 | 116 (22%) | 20 (15%) | 96 (24%) |  |
| 5-15 | 251 (47%) | 79 (59%) | 172 (43%) |  |
| Age (months) | 48 (18, 108) | 84 (23, 132) | 43 (17, 96) | **<0.001** |
| Sex female | 259 (49%) | 60 (46%) | 199 (50%) | 0.4 |
| *Missing* | *2 (0.4%)* | *2 (1.5%)* | *0* |  |
| TB history present | 30 (5.6%) | 10 (7.5%) | 20 (5.0%) | 0.12 |
| *Missing* | *3 (0.6%)* | *2 (1.5%)* | *1 (0.3%)* |  |
| TB contact present | 168 (32%) | 61 (46%) | 107 (27%) | **<0.001** |
| *Missing* | *11 (2.1%)* | *0 (0%)* | *11 (2.8%)* |  |
| Children with SAM | 331 (62%)* | 78 (60%) | 253 (63%) | 0.51 |
| *Missing* | *3 (0.6%)* | *3 (2.3%)* | *0* |  |
| Children with HIV | 93 (17%)** | 57 (43%) | 36 (9.0%) | **<0.001** |
| *Unknown* | *1 (0.2%)* | *0* | *1 (0.3%)* |  |
| CD4 <200 cells/mm^3$^ | 26 (34%) | 24 (44%) | 2 (9.1%) | **0.004** |
| *Missing* | *17 (18.3%)* | *2 (3.5%)* | *15 (41.7%)* |  |
| ART status^$^: on ART | 40 (43%) | 34 (60%) | 6 (16%) | **<0.001** |
| *Missing* | *1 (1.1%)* | *0* | *1 (2.7%)* |  |
| TB diagnosis |  |  |  | 0.3 |
| Confirmed | 85 (16%) | 26 (20%) | 59 (15%) |  |
| Unconfirmed | 238 (45%) | 60 (45%) | 178 (45%) |  |
| Unlikely | 210 (39%) | 47 (35%) | 163 (41%) |  |
| TB type^#^ |  |  |  | **0.007** |
| Disseminated | 92 (28.5%) | 24 (27.9%) | 68 (29.7%) |  |
| EPTB | 70 (21.7%) | 9 (10.5%) | 61 (25.7%) |  |
| PTB | 161 (49.8%) | 53 (61.6%) | 108 (45.5%) |  |
| Cough | 399 (75%) | 110 (83%) | 289 (72%) | **0.016** |
| Tachypnoea | 19 (3.6%) | 11 (8.3%) | 8 (2.0%) | **0.002** |
| Hypoxemia (SPO2 <92%) | 50 (9.4%) | 46 (35%) | 4 (1.0%) | **<0.001** |
| Fever | 440 (83%) | 92 (69%) | 348 (87%) | **<0.001** |
| Weight loss present | 374 (70%) | 8 (6.1%) | 366 (92%) | **<0.001** |
| *Unknown or missing* | *140 (26%)* | *125 (94%)* | *15 (3.8%)* |  |
| Gibbous | 28 (5.3%) | 17 (13%) | 11 (2.8%) | **<0.001** |
| Lymph nodes | 64 (12%) | 11 (8.3%) | 53 (13%) | 0.13 |
| Subacute meningitis | 5 (0.9%) | 1 (0.8%) | 4 (1.0%) | >0.9 |
| Abdomen distended | 28 (5.3%) | 6 (4.5%) | 22 (5.5%) | 0.7 |
| Diarrhoea | 49 (9.2%) | 0 (0%) | 49 (12%) | **<0.001** |
| Painless enlarged joints | 8 (1.5%) | 6 (4.5%) | 2 (0.5%) | **0.004** |
| Pleural effusion | 7 (1.3%) | 7 (5.3%) | 0 (0%) | **<0.001** |
| Other EPTB signs | 6 (1.1%) | 0 (0%) | 6 (1.5%) | 0.3 |
| No signs of EPTB | 345 (65%) | 94 (71%) | 251 (63%) | 0.10 |

Notes: *70/331 (21%) children with SAM were living with HIV. **70/93 (75%) children with HIV had SAM. ^$^Excluding data of children without HIV. ^#^ Excluding data of children with unlikely TB.

**Supplementary figure 1:** Venn’s diagram on concordance of Xpert MTB/RIF Ultra positivity per sample

**Supplementary table 2:** Description of cases diagnosed by Xpert MTB/RIF Ultra on stool and/or urine (gold standard negative)

| **Sex** | **Age** | **TB contact** | **Nutritional status** | **HIV status** | **Clinical presentation** | **Comments** | **Final Diagnostic** |
| --- | --- | --- | --- | --- | --- | --- | --- |
| Male | 7 yo | Yes | No | Negative | Cough, hypoxia, gibbous for 29 days | Admitted in Emergency room | PTB+EPTB |
| Female | 4 yo | No | SAM | Negative | Fever, acute diarrhoea, gibbous for 16 days | Admitted in ITFC | EPTB |
| Female | 13 yo | Yes | MAM | Negative | Fever and lymph nodes for 21 days | Followed in OPD  *Patient excluded from the analysis as GS was not obtained | EPTB |
| Male | 15 yo | No | No | Negative | Fever and gibbous for 36 days | Followed in OPD | EPTB |
| Male | 2 yo | Unknown | SAM | Negative | Fever and cough for 15 days | Xpert-Ultra in urine trace  Admitted in Pediatric ward | PTB |
| Male | 3 yo | Yes | MAM | Negative | Fever and cervical lymph nodes for 240 days | Followed in OPD | EPTB |
| Female | 16 mo | Unknown | MAM | Negative | Fever and abdomen distended 60 days | Xpert-Ultra on stool trace  Admitted in ITFC | EPTB |
| Male | 17 mo | No | SAM | Negative | Cough, fever, and diarrhoea for 21 days | Xpert-Ultra in urine trace  Admitted in ITFC | PTB+EPTB |
| Female | 5 yo | Yes | No | Negative | Cough and fever for 30 days | Xpert-Ultra on stool trace  Followed in OPD | PTB |
| Male | 18 mo | No | MAM | Negative | Fever and abdomen distended 90 days | Xpert-Ultra on stool trace  Admitted in ITFC | EPTB |

**Supplementary table 3:** Xpert MTB/RIF Ultra positivity rate per type of sample

| **Type of sample** | **Number of**  **sample**  **tested** | **TB Negative;**  **N (%)** | **TB Positive;**  **N (%)** | **Trace;  N (%)** | **Positive**  **or trace;**  **N (%)** |
| --- | --- | --- | --- | --- | --- |
| **Respiratory samples** | 500 | 451 (90.2) | 44 (8.8) | 5 (1) | 49 (9.8) |
| Gastric aspiration | 303 | 275 (90.8) | 26 (8.6) | 2 (0.7) | 28 (9.2) |
| Spontaneous sputum | 130 | 115 (88.5) | 13 (10) | 2 (1.5) | 15 (11.5) |
| Naso-pharyngeal aspirate | 63 | 57 (90.5) | 5 (7.9) | 1 (1.6) | 6 (9.5) |
| **Extra pulmonary samples** | 60 | 39 (65) | 17 (28.3) | 4 (6.7) | 21 (35) |
| Lymph node | 46 | 28 (60.9) | 15 (32.6) | 3 (6.5) | 18 (39.1) |
| Pus or tissue | 6 | 4 (66.7) | 2 (33.3) | 0 (0) | 2 (33.3) |
| Pleural effusion | 4 | 3 (75) | 0 (0) | 1 (25) | 1 (25) |
| CSF | 2 | 2 (100) | 0 (0) | 0 (0) | 0 (0) |
| Ascetic fluid | 1 | 1 (100) | 0 (0) | 0 (0) | 0 (0) |
| **Stool** | 524 | 512 (97.7) | 9 (1.7) | 3 (0.6) | 12 (2.3) |
| **Urine** | 493 | 451 (91.5) | 28 (5.7) | 14 (2.8) | 42 (8.5) |
