# Supplementary figures and images for: Xpert-Ultra Assay in Stool and Urine Samples to Improve Tuberculosis Diagnosis in Children: The Médecins Sans Frontières Experience in Guinea-Bissau and South Sudan
Source: Open Forum Infect Dis. 2024 May 2;11(5):ofae221. doi: 10.1093/ofid/ofae221 (PMC11119760; doi:10.1093/ofid/ofae221)

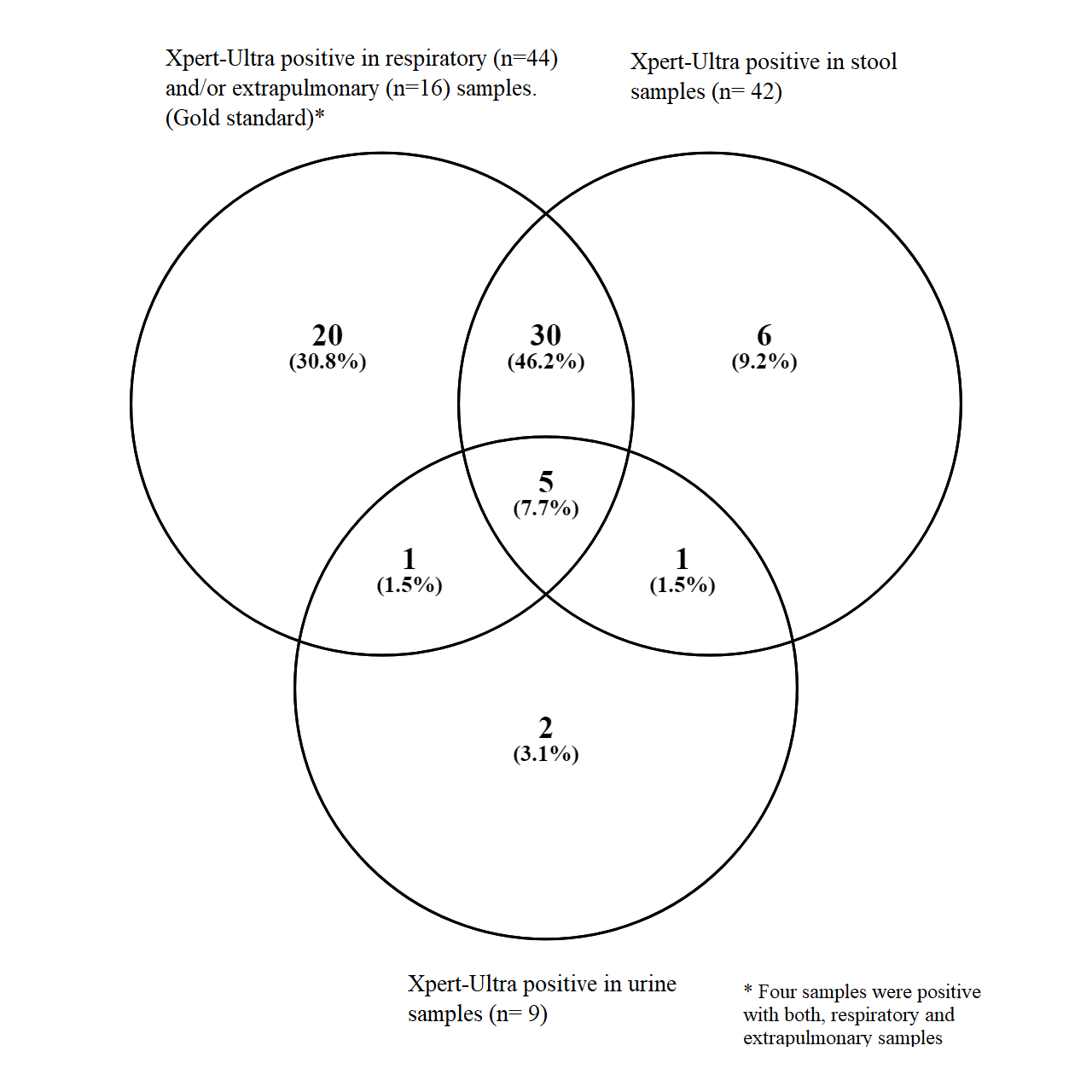

Supplement: ofae221_Supplementary_Data [file ofae221_supplementary_data.zip › Supplementary figure 1_050424.tif]
